# Beer Potomania: A View on the Dynamic Process of Developing Hyponatremia

**DOI:** 10.7759/cureus.3024

**Published:** 2018-07-22

**Authors:** Ratna Joshi, Shyan-Yih Chou

**Affiliations:** 1 Internal Medicine, Wycoff Heights Medical Center, New York, USA; 2 Nephrology and Hypertension, Brookdale University Hospital and Medical Center, New York, USA

**Keywords:** beer potomania, low solute intake, hyposthenuria, vasopressin, solute free water clearance, pathophysiology, urinary dilution and concentration

## Abstract

Poor solute intake has been ascribed to hyponatremia seen in patients with beer potomania, an uncommon etiology of hyponatremia. Our current understanding of how hyponatremia develops in these patients is derived only from individual cases described in the literature. In these case reports, the pathophysiology of beer potomania is explained exclusively by the concept of solute-free water clearance in the kidney. Specifically, low solute intake reduces urinary excretion of osmoles, thereby capping a ceiling on the renal capacity of free water excretion. A positive water balance follows an excess of water intake, causing dilutional hyponatremia. We propose that further inquiry is needed to explain how water is retained by the kidney. From reviewing the clinical data of these case reports, it is evident that there is a broad range of urine osmolality, ranging from levels below to above plasma osmolality. This finding is consistent with a dynamic course of vasopressin secretion during the development of hyponatremia. Vasopressin raises epithelial permeability to water in the collecting duct; the amount of luminal osmoles then determines the osmotic gradient for water transport. At a certain degree of hyponatremia, vasopressin secretion may cease and profound water diuresis ensues. Unfortunately, the status of vasopressin release is rarely investigated. We propose that in patients with beer potomania detailed fluid balance studies, sequential observations of changes in urine and plasma osmolality corresponding to dynamics of vasopressin release would advance our understanding of its pathophysiology.

## Introduction and background

An excess of total body water is indicated clinically by hyponatremia, a very common electrolyte disorder, and carries serious clinical consequences [1]. It is highly prevalent in hospitalized patients, after surgery and among the elderly. Chronic hyponatremia can lead to neurocognitive dysfunction, coordination, and gait deficits, or mood disorders, while severe acute hyponatremia can cause brain edema, seizures or even death. Understanding correctly the underlying pathophysiology of hyponatremia is key to proper management of hyponatremia and resolution of its clinical manifestations. The pathophysiologic process of hyponatremia is complex, resulting from a wide range of clinical disorders. Over the past decades, a number of classifications and diagnostic algorithms have been developed to guide identification of the pathophysiologic process responsible for hyponatremia and to direct its appropriate management. Recently, clinical practice guidelines have been developed separately by American and European medical organizations to help diagnosis and treatment of hyponatremia [2-3]. To further simplify the approach to uncover the specific cause of hyponatremia among diverse etiologies, diagnostic algorithms have been recommended, the most popular one being based on volume status of hyponatremic patients [2]. Although diagnostic algorithms on hyponatremia have the practical appeal of rapid determination of its etiology, they are limited by the rigid rules of problem-solving. An example is the algorithm recently reported, in which diagnosis of hyponatremia caused by beer potomania or low dietary solute intake is to be considered when urine osmolality is less than 100 mOsm/kg [4]. Accepting this recommendation may be problematic because urine diluted to this level would indicate excretion of solute-free water (CH_2_O) and no net water retention. If so, how would hyponatremia occur? Other questions then follow as to the natural course of beer potomania. Is there a phase of positive water balance during the development of hyponatremia? Is arginine vasopressin released in the process of developing hyponatremia in patients with very low solute intake? Would some patients manifest free-water reabsorption (negative CH_2_O expressed by osmolality of urine exceeding that of plasma)? These questions prompt us to review cases of beer potomania reported in the literature, which we hope may provide some clues to answer these questions. 

## Review

Natural course and clinical features of beer potomania

The reports of hyponatremic patients described as beer potomania first appeared in the medical literature in the early 1970’s [5-6]. Over the following years, more than thirty five cases have been reported with all of them as individual case reports [5-26]. These patients were hospitalized for symptoms and signs of various neurological impairment, including unconsciousness, seizures, confusion, and agitation. Other less specific symptoms are described as weakness, forgetfulness, nausea, dizziness and tremors. These symptoms are attributed to hyponatremia. A history is typically obtained, revealing binge beer drinking and a poor oral intake of solutes. The serum sodium concentrations ranged between 97 and 134 mmol/L (median, 109 mmol/L) (Table 1). Other frequently encountered laboratory features are hypokalemia (serum potassium, 1.4 – 5.5 mmol/L; median, 3.2 mmol/L) and low blood urea nitrogen (BUN) levels (2-30 mg/dL; median, 6 mg/dL). After receiving a solute load, these patients would undergo a brisk diuresis, resulting in rising serum sodium concentrations and ultimately correction of hyponatremia. The solute load is typically provided with intravenous administration of saline or oral feeding of proteins, which generate urea (approximately 50 mmol from metabolism of 100 g of proteins). Some authors emphasize the importance of hyposthenuria (urine osmolality <100 mOsm/kg) when considering the diagnosis of beer potomania [4]. In contrast, Sanghvi et al. [16] reviewed 22 cases of beer potomania in the literature and identified that hyposthenuria was not a consistent finding. These authors caution against inclusion of hyposthenuria in the diagnostic criteria for beer potomania to avoid erroneous dismissal of this diagnosis.

**Table 1:** Summary of Beer Potomania Studies. Abbreviations: BUN, blood urea nitrogen; N/A, not available; #, ADH (Antidiuretic hormone) was below the detection level; @, ADH was 49.9 pg/mL (normal range: 1-5 pg/mL); *, serum osmolality calculated as (2 x sodium) +10.

Author (Year)	Patients no.	SERUM				URINE	
		Sodium (mmol/L)	Potassium (mmol/L)	BUN (mg/dL)	Osmolality (mOsm/kg)	Sodium (mmol/L)	Osmolality (mOsm/kg)
Demanet et al. (1971) [5]	1	107	2.6	12	224*	N/A	N/A
	2	105	1.3	15	220*	N/A	N/A
	3	104	2.6	30	218*	N/A	N/A
	4	103	2.5	13	216*	N/A	N/A
	5	101	1.4	19	212*	N/A	N/A
	6	99	1.8	18	108*	N/A	N/A
	7	98	4.4	30	106*	N/A	N/A
Gwinup et al. (1972) [6]	1	122	5.1	N/A	254*	N/A	N/A
Hilden et al. (1975) [7]	1	123	2.7	N/A	236*	N/A	N/A
	2	109	2.5	N/A	228*	N/A	79
	3	108	2.7	N/A	226*	N/A	69
	4	127	2.5	N/A	264*	N/A	N/A
	5	117	3.1	N/A	244*	N/A	N/A
Swenson et al. (1976) [8]	1	106	3.8	N/A	222*	5.6	199
Evans et al. (1985) [9]	1	118	4.1	2.3	246*	NA	N/A
Joyce et al. (1986) [10]	1	110	3	Normal	230*	14	N/A
Fenves et al. (1996) [11]	1	121	3.6	4	252*	1	50
	2	97	3.6	2	204*	12	338
Kelly et al. (1998) [12]	1	109	3.6	16	228*	<10	340
Thaler et al. (1998) [13] #	1	134	4	6	278*	16	93
Hettema et al. (1998) [14]	1	124	5.5	5.2	286	27	126
Leens et al. (2001) [15]	1	97	2.1	14	204*	N/A	N/A
Sanghvi et al. (2006) [16]	1	100	2.7	4	210*	53	218
	2	104	4.3	7	218*	10	547
Nagler et al. (2006) [17]	1	117	3.3	6	225	146	390
Dickson et al. (2010) [18]	1	104	N/A	N/A	236	N/A	216
Bhattarai et al (2010) [19]	1	107	4	3	284	6	383
McGraw et al. (2012) [20]	1	105	N/A	N/A	225	16	75
Ko SH et al. (2014) [21] @	1	113	3.4	6.2	233	122	344
Cho YS et al. (2014) [22]	1	112	2.4	9.6	240	N/A	370
Pallavi (2015) [23]	1	119	2.8	N/A	277	8	79
Srisung et al. (2015) [24]	1	117	N/A	6	250	N/A	132
Kujubu et al. (2015) [25]	1	116	4.1	6	250	35	182
Lodhi MU et al. (2017) [26]	1	118	4	4	259	N/A	N/A
	2	106	4.6	9	232	19	159

Low intake of solutes and development of hyponatremia

In humans, normal glomerular filtration function coupled with maximum urinary dilution (e.g. urine osmolality, 50 mOsm/kg) allows excretion of water as much as 10-20 liters a day when daily solute intake is adequate (600 – 900 mOsm). A sharp reduction in dietary solute intake would largely limit the capacity of the kidney to excrete water. With this limitation, ingestion of water in a quantity exceeding that of renal water excretion would lead to water retention and result in dilutional hyponatremia. Hyponatremia seen in beer potomania or other situations of low dietary solute intake has been exclusively explained by the mathematical formula [27] as follows:

CH_2_O = V x [1- (UOsm/POsm)]            (1)

or CH_2_O = (solute excretion/UOsm) x [1-(UOsm/POsm)]            (2)

where CH_2_O is the volume of blood plasma that is cleared of solute-free water per unit time, also known as free water clearance, V is urine flow (volume of urine per unit time), and UOsm and POsm are urine and plasma osmolality, respectively. When UOsm is less than POsm, solute-free water is excreted and its quantity is proportionally increased following a rise in solute excretion. Equation (2) points out the critical role of solute excretion in describing how hyponatremia occurs in case of poor dietary solute ingestion. Low solute intake limits free water excretion. If water is ingested in an amount overwhelming the ceiling of free water clearance, positive water balance results in dilutional hyponatremia. Equation (2) also describes correction of hyponatremia by providing sufficient intake of solutes because an increase in solute excretion would raise the capacity of free water clearance. 

In all case reports in the literature, the pathophysiology of hyponatremia seen in patients with beer potomania is explained by urinary water excretion limited by inadequate solute intake as summarized above. However, there is a conspicuous lack of discussion on how water is retained to result in hyponatremia. The recommendation of hyposthenuria (Uosm <100 mOsm/kg) as a diagnostic feature of beer potomania could appear as a conundrum: hyponatremia occurs in the face of excretion of free water. It could be argued that free water excretion being less than ingested water serves as a simple explanation for positive water balance. However, it is erroneous to assume that the excess water dilutes serum sodium concentrations by circumventing glomerular filtration. With this in mind, a brief review of the mechanism of urinary dilution and concentration is in order.

Urinary dilution and concentration in the distal nephron

**Figure 1:**
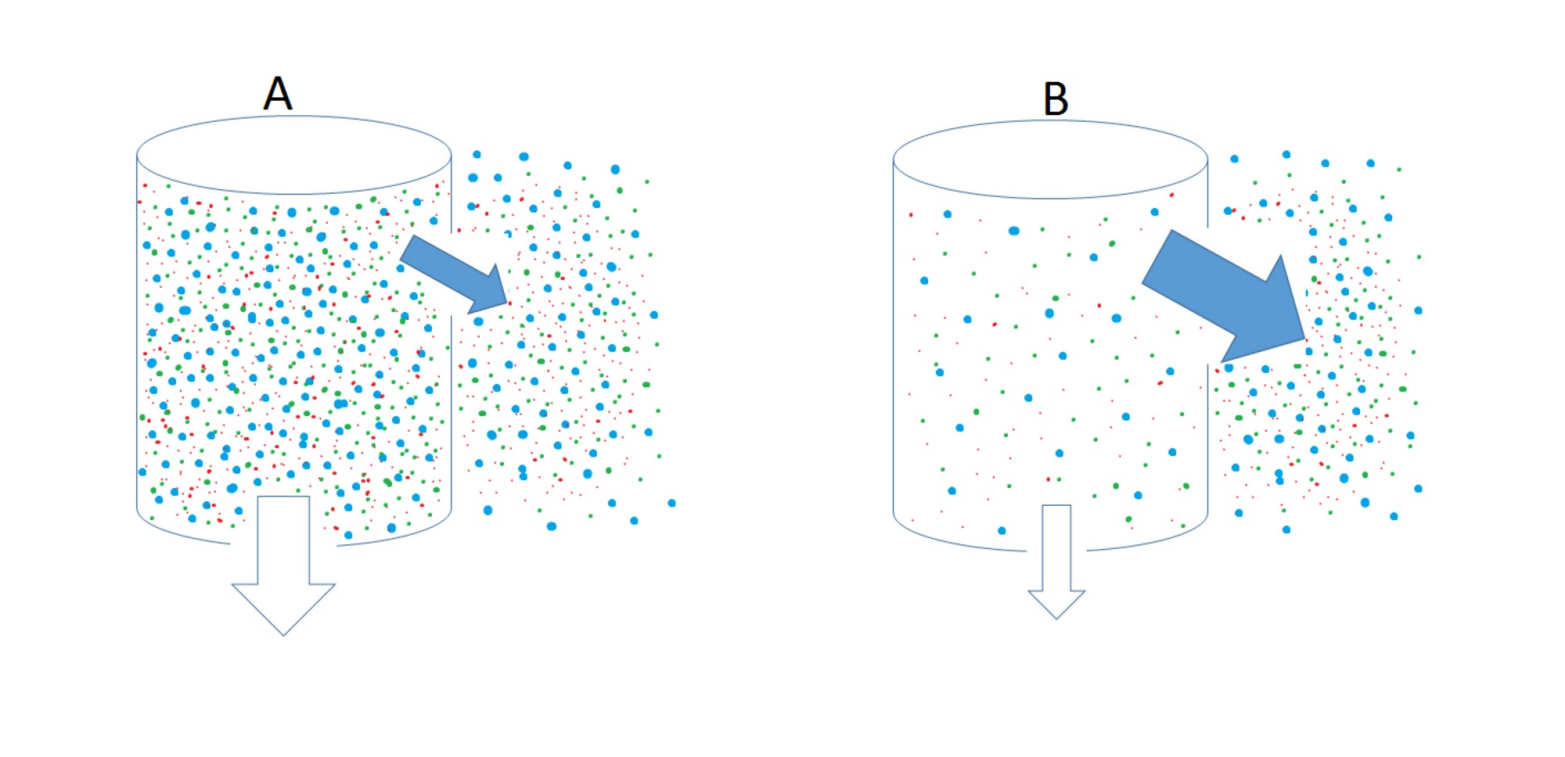
Schematic presentation of water transport from the lumen of the collecting duct into the interstitium in the cortex. The concentrations of small solutes are depicted by the density of particles, which in turn determines the osmotic pressure. The interstitium in the renal cortex is iso-osmotic to plasma while tubular fluid osmolality varies depending on solute concentrations. The solid arrows indicate water movement out of the lumen into the interstitium down the osmotic gradient. The open arrows show the flow of tubular fluid to the late collecting duct. Thicker arrows denote a higher volume of water movement than thinner arrows. The action of vasopressin renders the epithelium permeable to water. Water reabsorption across the tubular epithelium then relies on the osmotic gradient. Panel A shows a high luminal solute concentration impeding water reabsorption. Panel B shows a low luminal solute concentration enhancing water reabsorption.

Since plasma water must all be subjected to glomerular filtration, the ultrafiltrate water will be processed by various transport mechanisms along the proximal tubules, descending limb of Henle’s loop, and collecting duct. Thus the amount of water in the final urine amounts to that escaping tubular reabsorption. The water transported by the collecting duct determines both the urine quantity as well as the quality of being dilute or concentrated. As ultrafiltrate emerges from the thick ascending limb and enters the distal tubule in the cortex, urinary dilution continues to proceed. Tubular fluid entering the cortical collecting duct then becomes hypo-osmotic to the surrounding interstitium. Water reabsorption at this site is regulated by water permeability of the collecting duct epithelium. Vasopressin mediates trafficking of the water channel protein, aquaporin-2, to the apical plasma membrane and regulates water permeability [28]. In the presence of vasopressin, the collecting duct epithelium is highly permeable to water and water transport becomes dependent on the osmotic gradient between the luminal fluid and the surrounding interstitium. In the cortex, the interstitium is iso-osmotic whereas tubular fluid is hypo-osmotic to plasma. The gradient is thus favorable for water transport from the tubular lumen to the interstitium via aquaporin-2. The solutes in the tubular fluid are effective in exerting osmotic pressure in retarding water movement caused by osmotic gradients. Figure 1 illustrates the important role of tubular solutes in reducing water reabsorption along the cortical collecting duct. The osmolality of the interstitium is relatively constant, being iso-osmotic to plasma. The osmolality of the tubular fluid varies but is below the plasma level. The osmotic gradient between the tubular fluid and interstitium will thus be determined by solute concentrations of the tubular fluid. Low levels of solutes present in the tubular fluid would create a high osmotic gradient and increase water reabsorption in the collecting duct. It should be noted that urea constitutes a critical component of the small solutes in the collecting duct. In the cortex, collecting duct has a very low permeability property to urea, regardless of whether vasopressin is present or not [29]. In patients with beer potomania or poor solute intake, low blood BUN levels (Table 1) are consistent with the possibility that solutes present in the cortical collecting duct are inadequate to impede water reabsorption rendered by osmotic gradients.

**Figure 2:**
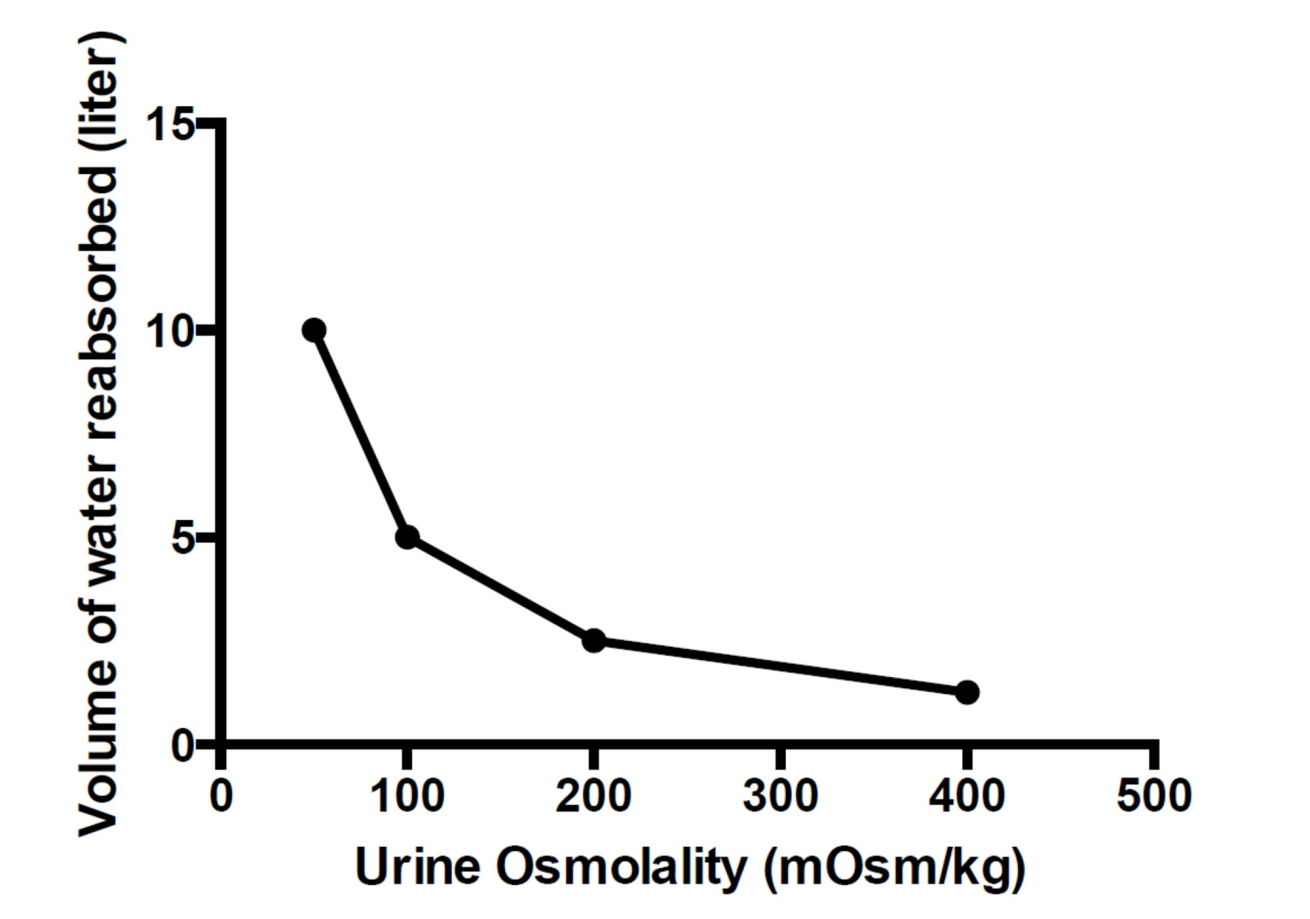
Volume of water reabsorbed in the cortical collecting duct estimated to double urine osmolality. Water reabsorption begins in the early cortical collecting duct with a hypothetical volume of 10 liters of tubular fluid and osmolality of 50 mOsm/kg. To concentrate the fluid to 100 mOsm/kg, 5 liters or half of its initial volume would be lost due to water reabsorption. For an additional doubling of urine osmolality to 200 mOsm/kg, the volume of water reabsorbed would be halved to 2.5 liters. A further decrease of water reabsorption to 1.25 liters would double urine osmolality to 400 mOsm/kg. In the process of urine concentration, a much greater quantity of water would be reabsorbed in the beginning than the late portion of the collecting duct.

Final urine is formed as tubular fluid flows from cortical to medullary collecting duct and its osmolality reflects the quantity of water that has been reabsorbed. The late distal convoluted tubule and cortical collecting duct are impermeable to urea. Under the action of vasopressin, water reclamation at these nephron sites would raise the urea concentration and tubular fluid osmolality. The amount of water transported out in relation with urine osmolality could be roughly estimated. This estimation suggests that more water is reabsorbed in the early than the late collecting duct as explained by the following example. At some point in the late distal tubule or early cortical collecting duct, urinary dilution mechanisms operate to makes tubular fluid osmolality as low as 50 mOsm/kg. To concentrate the fluid to 100 mOsm/kg, half of its volume would be lost due to water reabsorption. Figure 2 depicts the volume of water reabsorbed in the early collecting duct in order to double the urine osmolality. For example, at the starting point, 10 liters of fluid with an osmolality of 50 mOsm/kg collectively appear in the collecting duct. A volume of 5 liters of water needs to be reabsorbed to double the urine osmolality to 100 mOsm/kg. For an additional doubling of urine osmolality to 200 mOsm/kg, the volume of water reabsorbed would be halved to 2.5 liters. A further decrease of water reabsorption to 1.25 liters would double urine osmolality to 400 mOsm/kg. Thus in the process of urine concentration, quantitatively a much larger amount of water would be reabsorbed in the beginning than the late portion of the collecting duct. Over six decades ago, Berliner and Davidson [30] expressed a view that has long been overlooked: the function of vasopressin to cause the excretion of a hypertonic urine is much less important than preventing the excretion of a dilute urine. In the case of beer potomania, it is not necessary to excrete a hypertonic urine to indicate substantial water reabsorption in the collecting duct. The urine osmolality in patients with beer potomania could be hypo-osmotic or approach iso-osmotic while water is reabsorbed in the cortical collecting duct insofar as its epithelial water permeability allows water moving into the interstitium down the osmotic gradient. A steeper osmotic gradient would be maintained due to lower solute contents in the tubular fluid to favor water reabsorption.

Diversity of urine osmolality in patients with beer potomania. 

**Figure 3:**
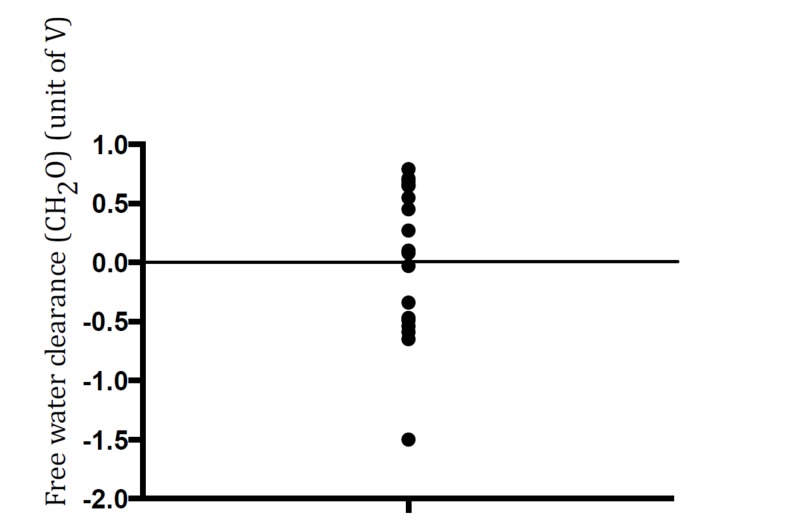
Free water clearance (CH2O) calculated for the patients reported in the literature. Solute-free water is calculated by the following formula, CH_2_O = V x [1- (UOsm/POsm)], where V is urine volume; UOsm and POsm are urine and plama osmolality, respectively. Since urine volume (V) is not reported in these case reports, the unit of CH_2_O is expressed as unit volume of V. Each dot represents a single case reported in the literature. CH_2_O varies from positive to negative values; a positive value indicates free water excretion and a negative value reflects water reabsorption. Alternatively, the electrolyte-free water clearance can be used to examine alterations in water excretion [13, 27]. In this approach, Uosm is replaced by the sum of urine sodium and potassium concentrations (UNa and UK, respectively) and Posm by serum sodium concentration (SNa) so that CH_2_O = V x {1- (UNa + UK)/SNa}. However, UK is not provided in the reported cases, thus precluding the calculation of CH_2_O in this way.

Urinary osmolality provides critical information about the status of vasopressin release. Among hyponatremic patients due to beer potomania or poor dietary solute intake, only single values of urine osmolality were reported after they sought medical care. Since no studies have been conducted on this subject, these individual case reports form the basis for the clinical and diagnostic characteristics. Despite the lack of studies, the individual data on urine osmolality can still serve as footprints to offer a glimpse into the dynamic process of vasopressin secretion during the development of hyponatremia. Figure 3 depicts individual values of CH_2_O approximated from the urine and serum osmolality described in these case reports. Urine volume is represented by the term 'V' because its exact value is not reported. A positive CH_2_O denotes excretion of free water and a negative value reflects water reabsorption along the collecting duct. Figure 3 displays a broad array of free water values, ranging from positive to negative ones. The diverse CH_2_O values may afford a platform to postulate that vasopressin secretion occurs at some stage during the development of hyponatremia. Nonosmotic stimuli for vasopressin secretion may be present in these patients, including nausea, abdominal distention or other stress. Unfortunately, plasma vasopressin levels have rarely been determined in these reported cases. In our search, we have identified only two cases in which plasma vasopressin levels were reported. In one case, the level was not detectable [13], whereas a high level was measured in the other case [21]. These divergent values support the possibility of the dynamic nature of vasopressin secretion during the clinical course of hyponatremia. Retention of water progressively lowers the plasma osmolality to a level sufficient to stop vasopressin secretion. This perhaps occurs in some patients, who are excreting highly dilute urine at the time of clinical evaluation.

An additional limitation imposed by the individual case reports is that fluid balance studies have not been performed in these patients except in one. Gwinup et al. [6] conducted a balance study in a patient with beer potomania while the patient was hospitalized. During the study, the patient drank more than 5 liters of beer daily for seven days, resulting in a positive water balance amounting to more than 1 liter per day. This was accompanied by progressive weight gains, lowering of serum sodium, and chloride concentrations as well as rising urine osmolality to over 800 mOsm/kg. Upon cessation of beer drinking, water retention ended, urine became hypo-osmotic, weight loss occurred, and correction of hyponatremia ensued. Although plasma vasopressin was not measured in this patient, this course of changing serum sodium concentration and urine osmolality is consistent with turning on and off of vasopressin release.

Collectively, the hyponatremia in patients with beer potomania arises from a constellation of factors that determine serum sodium concentrations. In addition to low solute intake, these patients consume a large quantity of water to exceed the renal capacity of water excretion, resulting in a positive water balance. Underneath the defective water excretion are the impacts of vasopressin and insufficient solutes to withstand the action of osmotic gradients in favor of water reabsorption in the collecting duct. Unfortunately, the status and dynamics of vasopressin release due to osmotic or nonosmotic regulation has not been investigated in beer potomania and remain speculative.

## Conclusions

Hyponatremia due to beer potomania or other forms of poor dietary solute intake is described only in individual case reports. In these reports, the concept of free water clearance has exclusively been applied to explain how renal water excretion is restricted by low solute excretion. We propose that further inquiry is required regarding how water reabsorption in the collecting duct is augmented by steep osmotic gradients created by paucity of solutes in the luminal fluid. Our proposal invokes the necessity of vasopressin to facilitate this transport. Unfortunately, no studies have been designed to gain a deeper insight into the pathophysiology of this uncommon syndrome of hyponatremia. In particular, the dynamic nature of vasopressin secretion in these hyponatremic patients is largely unknown. Specifically, vasopressin release into systemic circulation needs to be examined in the context of its relationship with plasma osmolality and extracellular fluid volume as well as nonosmotic stimuli for its release. Detailed fluid balance studies are also needed, accompanied by sequential observations of changes in urine and plasma osmolality to correspond to dynamics of vasopressin release.
